# Leveraging global investments for polio eradication to strengthen health systems’ resilience through transition

**DOI:** 10.1093/heapol/czad093

**Published:** 2024-01-23

**Authors:** Fabrizio Tediosi, Simone Villa, Darcy Levison, Ebru Ekeman, Claudio Politi

**Affiliations:** Swiss Tropical and Public Health Institute, Kreuzstrasse 2, Allschwil 4123, Switzerland; University of Basel, Petersplatz 1, P. O. Box 4001, Basel, Switzerland; Centre for Multidisciplinary Research in Health Science (MACH), University of Milan, Via Festa del Perdono, 7, Milano, 20122, Italy; Polio Transition Programme, World Health Organization, 20 Avenue Appia, 1211 Geneva, Switzerland; Polio Transition Programme, World Health Organization, 20 Avenue Appia, 1211 Geneva, Switzerland; Polio Transition Programme, World Health Organization, 20 Avenue Appia, 1211 Geneva, Switzerland

**Keywords:** Polio eradication, polio transition, health systems resilience, global health financing

## Abstract

Since the launch of the Global Polio Eradication Initiative in 1988, more than US$20 billion has been invested globally in polio eradication. The World Health Organization and its partners are currently supporting Member States to transition the functions used to eradicate polio to strengthen their health systems. This study analyses global polio activities through the lens of health systems and the Common Goods for Health (CGH). Polio activities include key health system functions such as surveillance and response systems and immunization, which are essential to maintaining resilient health systems. They also support essential functions such as policy development, planning, training and capacity building, which are often underfunded in many countries. To improve overall resilience, it is critical to continue to integrate these functions into local health systems so that the capacity built through the polio eradication programme can be used for broader public health purposes. It is vital that this integration process be tailored to each country’s unique health system context, rather than using a one-size-fits-all approach. While integration of all polio activities into local health systems is ideal, the transition to domestic financing may be coordinated with other global health financing mechanisms. This would reduce funding fragmentation and transaction costs, and allow for a focus on health system functions as a whole rather than just disease-specific efforts. The transition to domestic financing of polio activities could be staggered, prioritizing the transition to domestic funding for activities with limited global externalities, while seeking longer-term external funding for those that are global CGH.

Key messages

- The experience of the polio eradication initiative and its transition towards domestic financing is an interesting case of donor-funded assets and infrastructures requiring transition to new funding mechanisms and integration in local health systems.
- Polio activities intersect with key health system functions such as surveillance and response systems and immunization, which are vital for maintaining resilient health systems. These are global public goods with expected large global externalities and socio-economic benefits that would require long term funding at the global level.

## Introduction

The Coronavirus (COVID-19) pandemic has underscored the imperative of tackling transnational threats through public health investments, commonly known as global common goods for health (CGH)(Yamey *et al.*, 2019; Yazbeck and Soucat, 2019; WHO, 2021). Nevertheless, the evolving global socio-economic landscape and the political priorities of high-income countries have prompted donors to redirect their attention towards fostering sustainability and transitioning to domestic funding (Shroff *et al.*, 2022b). It is, therefore, crucial to rethink Development Assistance for Health (DAH), which has been focused on specific diseases, by aligning with the overarching goal of strengthening health systems to effectively address future threats (Marten *et al.*, 2022; Shroff *et al.*, 2022a).

Polio eradication has been an unwavering commitment on the global health agenda for several decades (WHO, 1988). Notwithstanding the remarkable progress made over these years, the story of polio eradication serves as a poignant reminder of the immense challenges associated with eradicating infectious diseases, despite substantial global investment. Since the establishment of the Global Polio Eradication Initiative (GPEI) in 1988, from resolution no. 41.28 of the World Health Assembly (WHA), the incidence of poliomyelitis decreased by 99.9% as of 2021. Furthermore, this global effort has succeeded in eradicating two out of three wild polioviruses (WPVs) in the last decade (i.e. WPV-2 and WPV-3 certified eradicated in 2015 and 2019, respectively). Driving this progress, the GPEI has been a vertical programme supported by external funds for a total budget exceeding US$20 billion since its establishment. The current Polio Eradication Strategy 2022–26 aims at permanently interrupting WPV1 transmission in the two remaining endemic countries (i.e. Afghanistan and Pakistan) and stopping circulating vaccine-derived poliovirus (cVDPV) spread, and eventually preventing polio outbreaks in non-endemic countries (Groce *et al.*, 2021). However, the trajectory of eradicating polio is challenged by the increase in cVDPV outbreaks, the result of insufficient polio vaccine coverage and the worsening security situation in many countries at high-risk for polio, exacerbated by disruptions to essential health services, particularly immunization, due to the COVID-19 pandemic (TIMB, 2021a).

Addressing these challenges will necessitate countries to adapt their strategies and secure sustainable external financing in the long run. To illustrate, it has been projected that the GPEI will require funding of more than US$4 billion to assist countries in their efforts to achieve certification of the worldwide eradication of polio by 2026 (WHO, 2022a). During the post-certification period, countries must secure the financing necessary to continue to sustain core polio essential functions.

As polio eradication nears, the World Health Organization (WHO), together with partners, is supporting Member States to successfully transition the assets, tools, expertise, methods and approaches established through the polio eradication programme, currently supported by GPEI, to strengthen national public health systems (WHO, 2017). The global framework for polio transition is the Strategic Action Plan on Polio Transition (2018–23), presented to the WHA in 2018. The Strategic Action Plan has three key objectives: 1. Sustaining a polio-free world after eradication of poliovirus; 2. Strengthening immunization systems, including surveillance for vaccine-preventable diseases (VPDs); 3. Strengthening emergency preparedness, detection and response capacity in countries (WHO, 2018). Assets and infrastructure established by GPEI provide support to public health systems beyond polio eradication. The support ranges from essential immunization, disease surveillance, outbreak preparedness and emergency response, to primary health care services. GPEI has been gradually reducing its contributions to national programmes (both financial and workforce), as countries are declared polio-free. The objective of polio transition is for national governments to take over the key functions previously supported by the GPEI network, managing them through the Ministry of Health and using domestic funding. The ultimate goal of polio transition is, therefore, to support the integration of the network, know-how and infrastructure established to eradicate polio into relevant parts of national health systems to achieve better health outcomes of that system and to sustain a polio-free world (WHO, 2018).

During the course of the Strategic Action Plan on Polio Transition (2018–23), it has become clear that many of those countries that have officially transitioned from GPEI need continued support from WHO and partners to maintain certain elements of key functions such as immunization, surveillance and outbreak response. Each country defines its own strategy through a ‘National Polio Transition Plan’ with specific transition phases gradually reducing reliance on external support. In this manuscript, we analyse the polio transition activities globally, focusing on 20 priority countries where, as of 2018, the majority of GPEI assets were located. We aim to determine how investments in polio eradication can be used to strengthen resilient health systems through a facilitated transition to both domestic funding and alternative external financing sources.

## Materials and methods

This study combines reviews of financial flows and polio transition performance indicators, with qualitative analysis of polio activities and assets. We first analysed and mapped the financial flows linked to the objectives of the Strategic Action Plan on Polio Transition and GPEI funding in the broader context of DAH in the polio transition priority countries.

Second, we analysed the progress of polio transition in priority countries using the indicators of the WHO polio transition monitoring and evaluation dashboard (WHO, 2022c). The monitoring and evaluation framework includes indicators along the ‘results chain’ of polio transition. The framework identifies for each objective, the input or activities required, the expected outputs and the outcome that should lead to impact. Objective 1, sustaining polio essential functions, requires maintaining polio expertise through integration, which is expected to lead to higher inactivated polio vaccine coverage and higher quality polio surveillance, and this in turn to no cases of paralysis due to VDPVs/WPVs. Objective 2 aims at strengthening immunization systems and VPD surveillance. Polio capacities should improve immunization systems and vaccine delivery, which should in turn support an increase in full routine vaccination coverage and increased VPD surveillance. This may result in a reduction in the number of outbreaks of VPD, with a consequent reduction in under-5 morbidity and mortality. Objective 3 focuses on strengthening emergency, preparedness, detection and response. Polio capacities should strengthen International Health Regulations’ core capacities in countries. This could lead to early outbreak detection through expanded surveillance, increased individuals receiving life-saving treatments, including vaccinations, and a strengthened ability to control infectious disease outbreaks. The development and endorsement of national polio transition plans is a key milestone for polio transition and a critical indicator for monitoring polio transition efforts. Of note, in the two countries where WPV1 is still endemic (i.e. Afghanistan and Pakistan) elimination is still the highest priority. Therefore, no national polio transition plan has been developed nor endorsed.

Third, we analysed the polio assets and activities in priority countries to assess how they interact, strengthen and/or depend upon the national and local health systems The analysis of the polio activities is based on several data sources: the mid-term review of the implementation of the Strategic Action Plan on Polio Transition (2018-2023) conducted in early 2022 (EuroHealthGroup, 2022); the national polio transition plans of the 20 polio transition priority countries and their comprehensive multi-year strategic plans for immunization; reports of the WHO country missions and of the Joint External Evaluations; Gavi, the Vaccine Alliance (Gavi), WHO, the Global Fund to Fight AIDS, Tuberculosis and Malaria (Global Fund) reports and articles published in peer-reviewed journals. We assessed the role of the different players in all polio functions and activities, highlighting where their contribution is significant. We reviewed the national polio transition plans of polio transition priority countries (Table S2 in supplementary materials) to identify which organizations, if any, hold responsibility for a function in the country. Each activity has been categorized as being held by: (1) government without any apparent external support; (2) government with some WHO support through GPEI or other funding; (3) significant WHO support through GPEI or other funding (i.e. United Nation’s International Children’s Emergency Fund (UNICEF), Gavi); or (4) significant WHO support through GPEI funding. We then assessed the level of integration of each polio function in domestic health systems, on the basis of how many activities are jointly implemented with local partners. We classified countries based on how many other diseases (e.g. measles) and/or areas of health (e.g. maternal and child health) the national polio transition plans were aiming to integrate or were already integrated. Each activity was, therefore, labelled as ‘Polio-specific’ if there was no integration with other activities, ‘Polio & 1’ if there was information regarding an intention to integrate polio with activities related to another disease/condition, ‘Polio & 2-3’ when foreseeing integration of polio with 2–3 diseases/conditions and ‘Polio & 4+’ in case the plan had integrated, or aimed at doing so, at least 4 diseases/conditions.

We then analysed how polio functions, activities and assets are related, support and depend on different components of domestic health systems. To this end, we used causal loop diagrams (CLDs) that help to map relationships between variables in a system and provide a visual representation of the system structure (De Savigny *et al.*, 2017; Cassidy *et al.*, 2022). We produced a simplified macro system-wide CLD that depicts how polio activities in their totality interact with national and local health systems. The CLDs were built starting from the analysis of the national transition plans of each polio transition country, then country-specific CLDs were combined in a step-wise process into a single CLD, to create a visual model of polio transition and the health system. Then we validated the CLD by reviewing the results of the mid-term review of the Strategic Action Plan, which included interviews with many stakeholders. To develop the CLD, we used Vensim® PLE (Version 9.3.5).

Lastly, we applied CGH lenses to polio transition activities and discussed the implications for a smooth transition to domestic funding. The WHO report ‘Financing Common Goods for Health’ defined CGH as ‘the core population-based functions or interventions that are essential to the health and well-being of entire societies.’(WHO, 2021). CGHs are either public goods^1^ or have large health or social externalities that can be national or transnational (Yamey *et al.*, 2019; Yazbeck and Soucat, 2019). CGH have another important characteristic: investing in such goods has large health and economic benefits. Therefore, they require public financing and action. However, at the national level, there is often inadequate demand for governments to prioritize investments in CGH, resulting in them being underprovided for by governments (Yazbeck and Soucat, 2019).

## Results

### Resource mobilization for health in polio transition priority countries

The extent to which countries can mobilize funding for polio essential functions depends on several factors, including the macro-economic outlook, levels of public spending on health, prioritization of the health budget and how efficiently resources are used. The 20 polio transition priority countries differ in their level of socio-economic development and in their capacity to mobilize financial resources for health (Table 1). Most countries have a low Human Development Index, as a result of short life-expectancy, low education attainment levels and economic development. The health expenditure ranges in all countries between 2.6% and 5.4% of GDP, and on a per capita basis between US$21 and 70 for those in the African Region (AFR), between US$23 and 202 for those in the Eastern Mediterranean Region (EMR) and from US$51 to 133 for those in the South-East Asia Region (SEAR). The role played by DAH in these countries varies. In the countries of AFR, it is estimated to account on average for 26.1% of Current Health Expenditure (CHE) with wide variation, while in countries in EMR and SEAR it accounts for 7.5% and 4.6% respectively (Table 2 and Figures S1-S2 in supplementary materials). In most low-income polio transition priority countries, DAH and specifically external funding for immunization will be needed for many years. In these countries, external funding for primary health care and immunization comes from a range of channels, including Gavi, the Global Financing Facility for Women, Children and Adolescents (GFF), Global Fund, UNICEF, multilateral development banks, direct bilateral support and GPEI (Saxenian *et al.*, 2022).

**Table 1.** Economic and health expenditure indicators in polio priority countries

		WHO regions		
	All PTP countries	AFR	EMR	SEAR
No. PTP countries	20	7	8	5
Population, million^‡^	2833	499	425	1909
GDP, US$ per capita^@^^,^*^,§^	1,559.8	1,138.8	1,640.8	2084
*mean (min-max)*	(516.9–3,978.6)	(524.7–2,063.6)	(516.9–3,978.6)	(1,127.1–3,894.3)
HDI^†,^^#^	0.557	0.493	0.565	0.637
*mean (min-max)*	(0.385–0.718)	(0.385–0.586)	(0.455–0.718)	(0.585–0.705)
CHE, as % of GDP^@^^,§^	4.6%	4.0%	6.6%	3.8%
*mean (min-max)*	(2.6–15.5%)	(2.9–5.4%)	(2.8–15.5%)	(2.6–5.2%)
CHE, US$ per capita^@^^,^*^,§^	63.2	42.4	85.5	74.7
*mean (min-max)*	(21.3–202.3)	(21.3–69.8)	(23.4–202.3)	(50.7–133.0)
DHE, as % of CHE^@^^,§^	85.3%	73.9%	92.5%	95.4%
*mean (min-max)*	(36.4–99.6%)	(36.4–95.8%)	(83.9–99.6%)	(89.5–99.5%)
OOPS, as % of CHE^@^^,§^	52.9%	47.9%	56.7%	56.7%
*mean (min-max)*	(23.2–74.8%)	(23.2–74.7%)	(44.8–74.8%)	(31.8–74.0%)
EXT, as % of CHE^@^^,§^	14.7%	26.1%	7.5%	4.6%
*mean (min-max)*	(0.4–63.6%)	(4.2–63.6%)	(0.4–16.1%)	(0.5–10.6%)

‡ Population estimates retrieved as of 1 January 2020, from United Nations, Department of Economic and Social Affairs, Population Division (2022).

@ Data for year 2020. ^†^ Data for year 2021.

* Figures in million current USD. ^§^ Four countries of WHO-EMR have no data: Somalia, Syria, Libya and Yemen.

# Somalia has no data. **Abbreviations**: AFR = African Region; CHE = Current Health Expenditure; DHE = Domestic Health Expenditures; EMR = Eastern Mediterranean Region; EXT = External Health Expenditures; GDP = Gross Domestic Product; HDI = Human Development Index; PTP = Polio transition priority; OOPS = Out-of-pocket spending; SEAR = South-East Asian Region; USD = United States Dollars; WHO = World Health Organization.

**Source**: WHO Global Health Expenditure Database. Available at https://apps.who.int/nha/database/ViewData/Indicators/en (last access on 15 December 2022) and UNDP website: Human Development Index (HDI). Available at https://hdr.undp.org/data-center/human-development-index#/indicies/HDI (last access on 15 December 2022).

**Table 2.** GPEI funding and status of Gavi contributions to polio transition priority countries

WHO region	Country	WB income level	Avg. GPEI funding per year^@^	Share of GGHE-D for 2020	Gavi recipient	Gavi graduation phase	The Global Fund recipient
AFR	Angola	Lower middle	6 836 400	1.0%	Yes	4	Yes
	Cameroon	Lower middle	6 213 000	2.5%	Yes	2	Yes
	Chad	Low	9 581 400	9.6%	Yes	1	Yes
	DRC	Low	23 721 400	7.5%	Yes	1	Yes
	Ethiopia	Low	16 281 200	1.7%	Yes	1	Yes
	Nigeria	Low	232 239 600	4.4%	Yes	3	Yes
	South Sudan	Low	9 910 600	35.5%	Yes	1	Yes
EMR	Afghanistan	Low	86 978 400	36.4%	Yes	1	Yes
	Iraq	Upper middle	5 363 400	0.1%	No	–	No*
	Libya	Upper middle	348 500	–	No	–	No
	Pakistan	Lower middle	232 239 600	8.0%	Yes	2	Yes
	Somalia	Low	15 609 600	–	Yes	1	Yes
	Sudan	Low	7 006 400	2.0%	Yes	2	Yes
	Syria	Low	3 783 600	–	Yes	1	No*
	Yemen	Low	4 007 400	–	Yes	1	No*
SEAR	Bangladesh	Lower middle	1 014 000	0.1%	Yes	2	Yes
	India	Lower middle	15 799 000	0.1%	Yes	3	Yes
	Indonesia	Lower middle	793 800	0.004%	No#	4	Yes
	Myanmar	Lower middle	1 256 600	0.2%	Yes	2	Yes
	Nepal	Lower middle	716 800	0.1%	Yes	1	Yes

^@^ Values refer to the period from 2018 and 2022. Libya has value only for years 2018 and 2019, while India does not have data for year 2022.

^#^Indonesia is fully self-financing and accessed the Pneumococcal Advance Market Commitment (AMC) price for pneumococcal vaccines.

* Last period with data of funds allocation is 2014–16. **Abbreviations**: AFR = African Region; DHE = Domestic Health Expenditures; DRC = Democratic Republic of Congo; EMR = Eastern Mediterranean Region; GGHE-D = Domestic General Government Health Expenditure; GPEI = Global Polio Eradication Initiative; PPR = Prevention, Preparedness, and Response; SEAR = South-East Asian Region; USD = United States Dollar; WB = World Bank; WHO = World Health Organization.

Sources: WHO Global Health Expenditure Database available at https://apps.who.int/nha/database/ Select/Indicators/en (last access on 15 December 2022), Gavi, the Vaccine Alliance—Annual Progress Report 2021 available at https://www.gavi.org/sites/default/files/programmes-impact/our-impact/apr/Gavi-Progress-Report-2021.pdf (last access on 15 December 2022), and the Global Fund Data Explorer website available at https://data.theglobalfund.org/ (last access on 12 January 2023). World Bank country classifications by income level: 2022–2023 available at https://datahelpdesk.worldbank.org/knowledgebase/articles/906519-world-bank-country-and-lending-groups.

### Financing of GPEI and the Strategic Action Plan on Polio Transition

**Figure 1.**
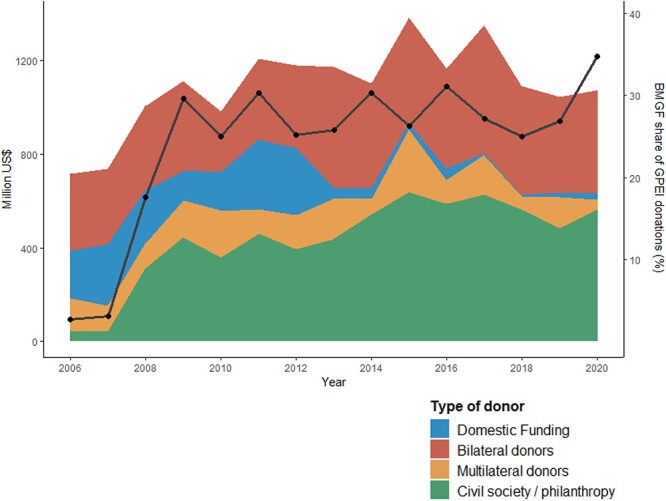
GPEI contributions according to donor type and the corresponding increase in funding from the Bill and Melinda Gates Foundation (years 2006–20). Solid black line represents BMGF share of GPEI donations

Both the GPEI strategy, which defines funding needs to eradicate polio, and the implementation of the Strategic Action Plan on Polio Transition, which defines resource needs for core essential functions like surveillance in countries that are transitioning from GPEI, are almost exclusively funded by external donors, although there is some variation in domestic contributions. Of note, GPEI increasingly relies on one major donor, namely the Bill & Melinda Gates Foundation, which in 2020 funded around 35% of the total GPEI budget (Figure 1). The level of GPEI funding varies by region and country, and it is related to both socio-economic development and the stage that countries have reached in the eradication process. GPEI funding accounts for a comparatively large percentage of the Domestic General Government Health Expenditure (GGHE-D) in some polio transition priority countries, such as South Sudan and Afghanistan, and for a sizable proportion of it in several other countries. In addition, all polio transition priority countries but Iraq, Libya and Indonesia are recipients of Gavi funds, while all but Iraq, Libya, Sudan and Syria receive funds from the Global Fund (Table 2). The fiscal impact of transitioning towards domestic funding for polio activities will thus be substantial for a number of countries. Mobilizing financial resources for polio activities is prone to challenges due to a relatively limited commitment of domestic resources from governments to take over funding for polio essential functions, fragmentation of funding and limited flexible funds due to strict donor rules for ensuring accountability and volatility of funds that makes predicting future funding hard.

**Figure 2.**
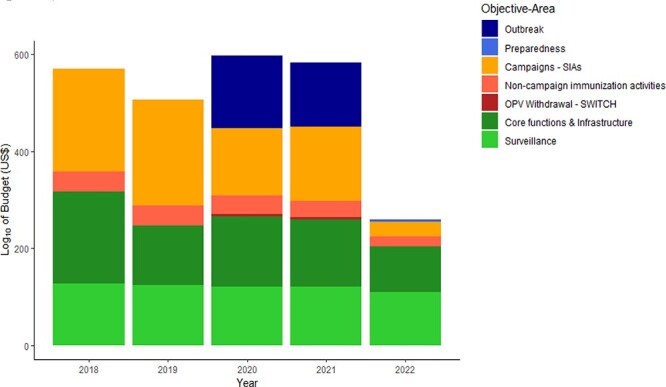
GPEI activity-specific budget (log10 transformed) during the period 2018 to 2022 in polio transition priority countries

GPEI funding for core essential functions to countries decreased from 2016 to 2021 across all polio transition priority countries. WHO is gradually increasing the amount of financing for core polio essential functions and is encouraging the integration of the functions into other programmes (e.g. immunization, primary health care, and health emergencies) and overall health systems. Transferring financing of polio essential functions in polio low-risk countries into the WHO Programme budget 2022–23 is a major achievement for polio transition. It should be noted, however, that the total GPEI budget remained essentially the same in 2022 as in 2021, even though it is now supporting only 13 high-risk countries while the WHO—through its programme budget—is committing to sustain polio essential functions in the remaining polio low-risk countries. Figure 2 shows the GPEI budget for specific activities over the last five years. Around half of the funds were used to implement immunization activities, a quarter for surveillance and a quarter for core functions and infrastructure. The GPEI budget for ‘Immunization’ covers the costs of oral polio vaccine procurement, campaign operational costs, campaign social mobilization costs and community-based vaccination costs. Funds for ‘Surveillance’ include costs for laboratory, technical assistance and other surveillance and running costs, while ‘Core functions & Infrastructure’ includes quality improvements, communications, community engagement and social mobilization costs and technical assistance. In 2021 and 2022, a substantial amount of funds were re-programmed to respond to the COVID-19 pandemic, although the actual contribution of polio staff to COVID-19 related activities is estimated to be larger (refer to Box S1 in supplementary materials).

The funding allocated by GPEI through the WHO primarily supports surveillance and core functions, including immunization activities. On the other hand, a larger proportion of the funds provided by UNICEF is directed towards immunization efforts and community engagement activities (refer to Figure 2 and Figure S3 in supplementary materials).

### Global polio transition progress and regional strategies

**Figure 3.**
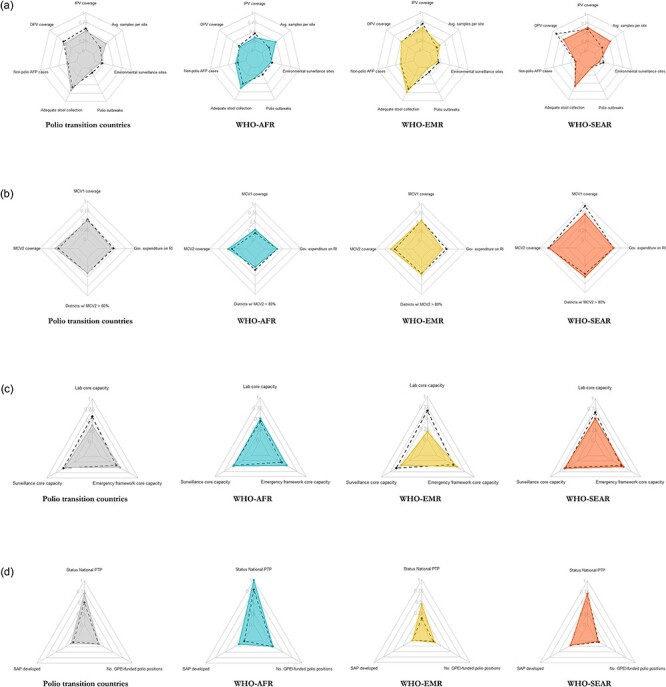
Polio Transition Programme’s M&E dashboard, comparison between 2018 ( dashed lines) and 2021 (polygon)

The polio transition monitoring and evaluation indicators show a mixed picture with regard to progress on polio transition. Regarding objective 1, from 2018 to 2021, the coverage of inactivated poliovirus vaccine has increased in most countries, and the quality of surveillance for acute flaccid paralysis (AFP) has remained relatively stable (Figure 3). AFP indicators show a positive outlook of stability and high performance across most polio transition countries except in WHO-AFR (EuroHealthGroup, 2022). Regarding objective 2, the average coverage with measles containing vaccine has been either relatively flat or declining, particularly during the period 2020–21, despite the increase in government expenditures on routine immunization in some priority countries and investments from Gavi (Figure 3). In most polio transition priority countries, except those in the WHO-SEAR, objective 2 indicators are still below the performance targets. Regarding objective 3, there have been promising improvements in all countries since 2018. The averages of three country self-assessed indicators on laboratory, surveillance and emergency framework core capacities, respectively, have generally increased over the period 2018–21.

The development of national polio transition plans has been highly WHO-led in some countries with the insufficient engagement of all key stakeholders and with the expectation that the government will implement the plans by using a combination of domestic and external funds (EuroHealthGroup, 2022). In the last few years, there has been a reduction in GPEI-funded staff aligned with the declining resources from GPEI. It has been estimated that the total number of WHO staff funded by GPEI decreased by 31% from 2016 to 2021, mainly in AFR (37%), while both EMR and SEAR had decreases of approximately 10%. The decline in the number of GPEI positions over the same time period in WHO headquarters was 6% (EuroHealthGroup, 2022). The expectation in several national polio transition plans is that the government will ultimately support the human resources that are required to implement polio essential functions.

WHO’s annual report on the implementation of the Strategic Action Plan on Polio Transition (2018–23) presented at the Executive Board’s 152nd session and updated for the WHA 76, provides an update on how polio transition is pursued in the different regions (WHO, 2023). In the AFR, 10 polio high-risk countries (refer to Table S1 supplementary material) continue to receive support from the GPEI. In the remaining 37 low-risk countries, surveillance and immunization activities have been fully integrated into broader public health functions, which continue to receive technical support from the WHO. In AFR, integrated public health teams are increasingly used to respond to other emergencies, leveraging the polio network and infrastructure. SEAR is the most advanced region with regards to polio transition. In the five polio transition priority countries of this region, the integrated network for surveillance and immunization originally set up to eradicate polio continues to strengthen immunization, measles and rubella elimination, surveillance for VPDs and health emergency response. The EMR, the region with the world’s two remaining polio-endemic countries, Afghanistan and Pakistan, is advancing the transition agenda in six priority countries. Two of the six transition priority countries in the region, Somalia and Yemen, are experiencing active outbreaks of cVDPV. In all countries, the surveillance network established for polio is supporting VPD surveillance and outbreak response.

### Integration of polio transition activities in national health systems

Polio activities cover five key functions that have a health system-wide impact: (1) integrated surveillance, (2) routine immunization, (3) outbreak and emergency response, (4) policy and planning and (5) training and capacity building. Integrated surveillance across diseases, syndromes and pathogens includes several subcomponents: (a) overall programme functions including data management and analysis, (b) peripheral surveillance, mainly focused on community-based surveillance and contact-tracing and collection of environmental samples and (c) laboratory functions for diagnostic confirmation of cases and environmental samples.

Each of these polio functions is implemented usually through a combination of domestic health systems and external support partners. Table 3 shows the institutions involved for each polio function. In most polio transition priority countries, the government plays a minor role in implementing most polio-related surveillance functions. Indonesia is the only country where the government leads the implementation of almost all polio activities. Nonetheless, there is variability in the degree of country government involvement in surveillance functions across the three WHO regions. Among countries in SEAR, most key activities are undertaken by country governments, with only a few activities where greater WHO support is provided. In EMR, most countries are implementing Integrated Public Health Teams, constituted by merging WHO country office staff from across different teams, especially the Polio Eradication Programme, Expanded Programme on Immunization and WHO Health Emergencies Programme fulfilling cross-team functions to provide integrated support to the country (Government of Sudan, 2021; Government of Syrian Arab Republic, 2021). In AFR, except in Angola, in most countries, key activities (e.g. peripheral surveillance) are performed entirely by WHO and/or GPEI.

**Table 3.** Summary of key polio functions by polio transition priority country and WHO region (excluding Afghanistan and Pakistan which are still wild poliovirus endemic)

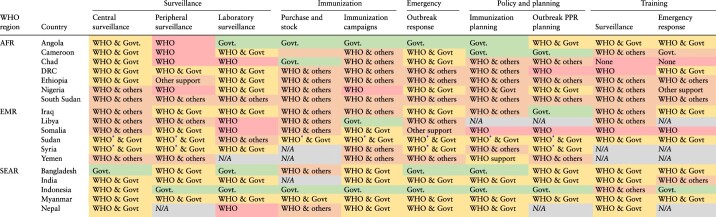

* Integrated Public Health Teams have been implemented. **Abbreviations**: AFR = African Region; DRC = Democratic Republic of Congo; EMR = Eastern Mediterranean Region; GPEI = Global Polio Eradication Initiative; PPR = Prevention, Preparedness, and Response; SEAR = South-East Asian Region; WHO = World Health Organization.

A similar regional pattern can be seen in the level of integration of polio activities in local health systems (Table 4). Polio activities are still vertical in most settings, although integration in countries’ health systems is increasingly happening. Polio transition countries have, in some cases, started the transition process by taking responsibility for some key polio activities and functions using domestic funding. This can be seen in Table 3 where the gradual transition is displayed as a colour shift from red (i.e. significant WHO support, through GPEI funding) to orange (i.e. WHO support, through GPEI or other funding) to yellow (i.e. Government, with some WHO support through GPEI or other funding) and, finally, to green (i.e. government). Countries in SEAR have more government-led activities (yellow) or without (green) external support from the WHO, while a few activities in EMR countries are entirely government-led. In AFR, most countries implement polio activities as stand-alone activities, in a more traditional ‘vertical’ manner. Overall, the two facing major challenges in terms of integration are peripheral surveillance and laboratory functions. Several, but not all, countries have a Polio Reference Laboratory.

**Table 4.** Summary of key polio functions with current or foreseen integration (based on countries polio transition SAPs) by priority country and WHO region

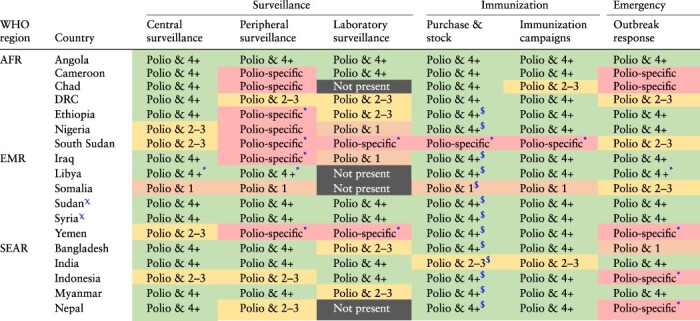

Afghanistan and Pakistan were excluded because still classified as wild poliovirus endemic countries. The integration of polio functions with health system ones is categorized as ‘**Polio-specific**’ if only covers polio functions, ‘**Polio & 1**’ if functions covering another disease (usually measles) or health areas (e.g. maternal health) has been integrated, ‘**Polio & 2-3**’ in case of 2–3 diseases/heath areas (not limited to VPDs) are covered, or ‘**Polio & 4+**’ if 4 or more diseases/health areas are integrated to polio. Other categories are ‘**not present**’ if the function is not present in the country or ‘**N/A**’ if there is no information available.

* In the Polio Transition Plans there is no detail on the level of integration of these functions. As those are certainly hold by the Polio Programme they have been labelled accordingly.

$ There is no information in the national polio transition SAPs on the level of integration of this activity. As the staff/department administering vaccine should also take care of vaccines stock management, the same level was attributed to this specific activity.

χ These countries have merged different programmes to create an Integrated Public Health Team to sustain the three functions of VPDs surveillance, routine immunization and outbreak response. **Acronym**: AFR = African Region; DRC = Democratic Republic of Congo; EMR = Eastern Mediterranean Region; GPEI = Global Polio Eradication Initiative; PPR = Prevention, Preparedness, and Response; SEAR = South-East Asian Region; WHO = World Health Organization.

The integration of polio activities in domestic health systems is pursued mainly by expanding staff terms of reference and reassigning staff to other functions/activities. In fact, polio staff members have always performed other non-polio-specific functions (e.g. routine immunization, VPD surveillance) to respond to local health needs. Integration is also happening through the contribution of polio staff to crucial cross-cutting functions, such as training and policy formulation. These functions are important for the successful implementation of immunization, disease surveillance and response systems that are key for pandemic preparedness.

The results of the stakeholders’ survey conducted during the mid-term review of the Strategic Action Plan for Polio Transition confirm these findings. Stakeholders who were interviewed indicated that programmatic and administrative functions are at an advanced stage of integration in most countries. However, they noted that integrating polio activities to local health systems is challenged by the physical separation of activities, donor fragmentation and power dynamics across stakeholders (EuroHealthGroup, 2022).

Despite the challenges associated with integration, polio activities make a valuable contribution to strengthening local health systems through a variety of means. It is therefore vital that the transition process is a success, to mitigate the risk of the systems built around polio activities collapsing when external support that has long sustained them is withdrawn.

**Figure 4.**
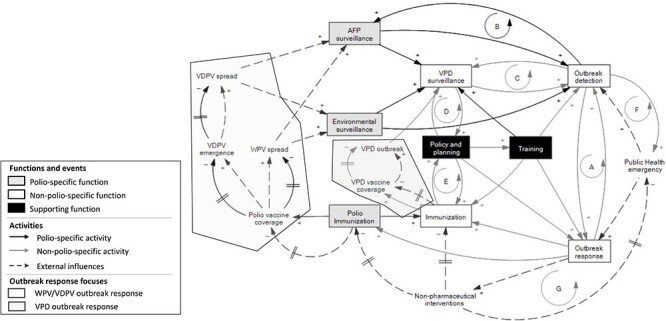
High level snapshot of Polio and Health System Causal Loop Diagram. Feedback loops from A to F are reinforcing loops, while loop G is a balancing one.

The CLD in Figure 4 provides a high-level snapshot of how polio activities interact with domestic health systems overall. The polio-specific functions and activities strengthen the whole system by contributing to disease surveillance, immunization, the emergency response system and to policy, planning and capacity building. Specifically, AFP surveillance and polio environmental surveillance—in the countries that have developed this capacity—may contribute to strengthening surveillance of other VPDs and non-polio outbreaks. Four out of five reports by the Transition Independent Monitoring Board (TIMB), which monitors progress towards polio transition, have underscored the polio surveillance system as a common good for health (TIMB, 2017a; 2017b; 2021a; 2021b). The laboratory capacity and field health worker networks established for AFP surveillance are increasingly used to detect other diseases. A stronger laboratory capacity supports both vaccine-preventable and infectious disease surveillance, and therefore the overall surveillance and emergency response capacity. Field health workers used for AFP surveillance often contribute to strengthening community-based surveillance and the essential immunization programme.

Polio immunization campaigns, particularly when integrated with the delivery of other vaccines and non-vaccine health interventions, have the potential to strengthen immunization programmes at both local and higher levels. However, the effectiveness of these campaigns hinges on their ability to consider and adapt to the intricate and dynamic nature of the health systems within which they are implemented (Neel *et al.*, 2021).

Health workers employed in polio campaigns often perform additional tasks for both routine immunization and other immunization activities. Overall, investments by the polio eradication programme may strengthen the vaccine supply chain, to the benefit of the essential immunization programme.

When an infectious disease outbreak is detected, countries develop and adopt outbreak-specific responses. Polio activities strengthen the capacity of the system to face the outbreak with personnel who can be quickly mobilized to provide technical support (refer to Figure 4, reinforcing loop A). For VPDs, polio staff can use the lessons learned from polio eradication to implement immunization campaigns to fill immunity gaps resulting from insufficient routine immunization (e.g. polio supplementary immunization activities) and enhanced surveillance such as contact tracing activities, for polio (Figure 4, reinforcing loop B) and/or other VPDs (refer to Figure 4, reinforcing loop C). If an outbreak evolves into an epidemic, especially when caused by non-VPDs, non-pharmaceutical interventions (e.g. isolation) can be adopted to stop the spread of the disease (refer to Figure 4, balancing loop G) besides countries creating/activating *ad-hoc* emergency teams for outbreak detection (refer to Figure 4, reinforcing loop F). However, this can delay immunization activities including campaigns for polio and other VPDs. If this happens and the epidemic does not quickly resolve, the diversion of resources to address the outbreak can result in decreasing levels of vaccine coverage for VPDs, including for polio. When the country still uses oral polio vaccines, this can lead to the emergence of VDPV which can eventually spread and cause AFP cases. Furthermore, if the epidemic affects polio-endemic countries (i.e. Afghanistan and Pakistan), the decrease in polio vaccine coverage can lead to increased risk of WPV spread within and beyond the country’s borders. In both cases, this would require a national and international response.

Information and evidence accrued from VPD surveillance (e.g. VPD burden, pathogen strain distribution, case characteristics especially vaccination status) and immunization (e.g. vaccine coverage, vaccine stocks) is used by decision makers to monitor health system needs, and develop, or adapt, vaccine and immunization policies (refer to Figure 4, reinforcing loops D and E). Decisions that can be informed range from immunization schedule optimization to programme planning to address coverage inequities by tailoring activities for those un-or under-immunized. There is also scope to use this information to set up or adjust training of staff working at different levels of the health system, from immunization and surveillance for polio and other VPDs, to outbreak detection and response planning, immunization logistics and cold chain management. Training and policy planning are important for the successful implementation of polio activities. The polio programme implements polio-specific training and policy planning activities that are, at least partially, overlapping with those of other infectious VPDs.

VPD surveillance and outbreak detection and response functions are strengthened by, and in some cases are developed from, polio surveillance (both AFP and environmental). There is strong potential for polio immunization to be integrated with other immunization activities (e.g. vaccine administration, social mobilizers and outbreak-led immunization campaigns). The case for integration is strengthened as having specific human resources handling different diseases in the context of multiple outbreaks (e.g. COVID-19 and subsequent emergence and spread of VDPVs) can weaken or delay a country’s response to public health emergencies.

### Polio transition phase-out strategy and global financing

Neither GPEI nor the WHO has set out a formal exit strategy for polio eradication. Specifically, GPEI has not defined phases for stopping support to polio low-risk countries, as opposed to the phase-out strategies of other financing mechanisms such as Gavi and the Global Fund (refer to Box S2 in supplementary materials). WHO has encouraged each country to define its own transition approach through national polio transition plans, which include specific phases and/or scenarios. Additionally, there is no post-transition support foreseen that will be funded by GPEI, although it is accepted that fragile countries will need continued support for specific functions, such as for disease surveillance. Given the interrelated challenges faced by polio transition countries of securing long-term global health investments, investing domestic resources in their health systems and protecting the wide health system contributions of polio activities, it is important to define a more structured approach to transition from donor to domestic or other sources of funding.

To this end, adopting a CGH lens allows us to identify key polio functions and activities that may require mid- and long-term global funding and those that should be prioritized first for transition to domestic funding. Polio eradication as a whole and population-based polio functions and activities that are being considered for transition have some features of CGH (Table 5). Polio eradication is a global public good that would have tremendous health and socio-economic benefits. A study conducted back in 2010 estimated incremental net benefits associated with polio eradication of US$51–59 billion (at 2021 US$) for the period 1988–2035, assuming eradication by 2012 (Tebbens *et al.*, 2010). A more recent analysis updated the estimates of the benefits of interrupting polio transmission by 2023 to US$30 billion (at 2021 US$) reflecting the delays in the eradication (Thompson *et al.*, 2022).

**Table 5.** Polio functions and activities viewed with Common Goods for Health lenses

			(Positive) Externalities			
Polio function	Polio activities	Global public goods	Global	National	Large health & economic benefits	Implications for Polio transition exit strategy
Policy & planning	Immunization planning			Yes		Domestic public funding^1^/Integrated in Gavi Strategy^3^
	Health emergency preparedness			Yes		Domestic public funding^1^
Training & capacity building	Surveillance & monitoring			Yes		Domestic public funding^1^
	Emergency assessment and response			Yes		Domestic public funding^1^
Integrated surveillance & monitoring	Central and community-based surveillance; AEFI surveillance	(Yes)	Yes	Yes	Yes	Long-term global support^2^/Integrated in Gavi Strategy^3^
	Laboratory (AFP environmental)	(Yes)	Yes	Yes	(Yes)	Long-term global support^2^
Immunization	Vaccines procurement and stock; cold chain management; service delivery		Yes	Yes	Yes	Integrated in Gavi Strategy^3^
Outbreak and Emergency response	Health emergency response	Yes	Yes	Yes	Yes	Long-term global support^2^

1 Activities that could be supported by public domestic funding; ^2^ Activities that could be supported globally to all LMICSs by global health financing mechanisms or institutions; ^3^ Activities that could receive support as per the Gavi phase out strategy.

**Abbreviations**: AEFI = Adverse Events Following Immunization; AFP = Acute Flaccid Paralysis. **Source**: authors analysis based on literature;.

Some of the activities of immunization programmes, such as surveillance, community engagement and public information campaigns, safety and quality regulations and protocols and overall vaccination policies, have the characteristics of CGH (Yamey *et al.*, 2019; WHO, 2021). Vaccination programmes in general can be considered CGH when they have large health or social externalities similar to the services that can reduce the spread of communicable diseases. Along these lines, polio vaccination can be considered a CGH due to large externalities linked also to health and socio-economic benefits of eradication. Polio activities can contribute to strengthening routine immunization programmes, which have large economic benefits, as shown by a recent study estimating a return for one dollar invested in vaccination against 10 pathogens in 94 low- and middle-income countries (LMICs) of US$19.8 from 2021 to 2030, including tangible and intangible benefits (Sim *et al.*, 2020). The transition to domestic funding of polio activities related to immunization could be more aligned with the criteria used by the Gavi phase-out strategy, at least in the countries that are still supported by Gavi. Polio activities that strengthen disease surveillance and response and broader epidemic/pandemic preparedness capacity, thus strengthening health system resilience, have large externalities and socio-economic benefits and contribute to the global public good of polio eradication. There is the potential to further integrate these activities into health systems, supported by longer term global funding in most countries through existing global health financing mechanisms for epidemic/pandemic preparedness capacity strengthening and also increased domestic public financing for sustainability. The polio activities related to policy and planning and those of training and capacity building are instrumental to strengthening the whole system. Nonetheless, their impact and externalities are more likely to be felt at the national level rather than at the global level. Therefore, these are the first activities that could be prioritized for transition from external to domestic funding in most countries (Table 5). The introduction of a co-financing mechanism could be explored further to incentivize domestic financing for activities during the polio transition process. Drawing inspiration from the experience of co-financing policies of Gavi and GFTAM, this approach would require countries to actively support and contribute to specific activities, thereby fostering greater ownership and accountability.

## Discussion

The remarkable progress achieved in recent years towards eradicating polio faces significant challenges due to worsening security conditions in high-risk nations, disruptions and economic impacts caused by the COVID-19 pandemic, and the limited resource allocation in certain countries for their health systems. At the same time, countries that have eliminated polio are transitioning away from GPEI resources to alternative funding mechanisms. To the best of our knowledge, this study is the first to consider polio transition in relation to the overall health system and CGH approach rather than simply as a financing issue. However, it is important to acknowledge several limitations associated with this study, primarily arising from the use of secondary data. Primary data, such as in-depth interviews with key informants using specifically designed tools to validate the study’s findings, could have bolstered the overall strength and reliability of our analysis. Despite these limitations, the findings of this study offer valuable insights that can guide policy decisions.

The successful transition from polio eradication to the long-term maintenance of countries’ polio-free status will require continuous commitments from polio transition priority countries and substantial external financial support over an extended duration. A swift transition to domestic funding alone does not appear feasible as it would place a significant financial burden on several countries and jeopardize the sustainability of eradication efforts.

In some countries, the transition process is still largely led by the WHO, with limited involvement from key stakeholders. However, in most countries, the process of incorporating polio-related activities into local health systems is ongoing, although the level of integration is still limited. This integration process should be tailored to the unique context of each country’s health system, rather than using a one-size-fits-all approach.

The resources and assets mobilized by polio eradication efforts, along with the technical assistance provided by the GPEI and the WHO to high-risk countries, can be further leveraged during the transition to enhance the resilience of health systems. Despite considerable variations among countries, polio activities are found to be interconnected with vital health system functions such as surveillance and response systems and immunization, in all 20 countries analysed. These functions are crucial for the development of resilient health systems; Figure 4, illustrating the polio and health system CLD, highlights the significance of polio activities as an integral component of various health system functions.

The COVID-19 pandemic has brought to light the valuable role of polio transition infrastructure and workforce in bolstering the resilience of health systems. This underscores the significance of fostering synergies and integration among disease-specific programmes, as illustrated in Box S1 of the supplementary materials. Studies have estimated that over 60% of the polio workforce dedicated at least 50% of their time to the COVID-19 response, further exemplifying their contribution (WHO, 2022b).

Polio activities not only help to eradicate the disease, but can also bolster essential health systems functions such as policy development, planning, training and capacity building. These are often neglected or underfunded in many countries. Ensuring continued integration of these functions into local health systems is crucial for enhancing the overall resilience of the healthcare system.

This study provides insights on the principles that should guide the implementation of the Strategic Action Plan on Polio Transition, as outlined in Table 5. It highlights that several polio-related activities have characteristics of global CGH and that investments in CGH can have benefits that extend beyond individual countries. However, it also notes that while individual countries have a role to play in financing these activities, additional funding is necessary. For example, investing in improved infectious disease surveillance and response systems in one country can greatly reduce the spread of an epidemic, but since many of the benefits of such improvements accrue to other countries, individual nations may prioritize the funding of activities that have a greater domestic impact. Given the risk of international spread of disease outbreaks, external support is justified to ensure that effective national surveillance programmes are adequately financed, with evidence suggesting that this will not displace dedicated national funding (Yamey *et al.*, 2019).

Based upon these factors, there is a distinction between transition towards domestic funding of polio activities and the process of integrating them in local health systems. While it is desirable to integrate all polio activities into local health systems, the transition to domestic funding could follow a different pathway and be coordinated with other global health financing mechanisms. The financial support to polio activities could be integrated, or at least coordinated, with that provided by other donors and financing mechanisms that are supporting immunization, such as Gavi, and pandemic preparedness, such as the Pandemic Fund established by the World Bank^2^. Although such an approach would be challenging to implement, it would reduce funding fragmentation and transaction costs, allowing efforts to be focused on the health system functions as a whole rather than just disease-specific efforts. Additionally, it could potentially feed into a coordinated resource mobilization strategy across WHO and major donors, towards securing flexible funds and implementing a holistic approach to using external funds for strengthening health systems. Considering the challenges faced by countries, the transition towards domestic funding for polio activities could be staggered. This would allow, for instance, for activities with limited global externalities to be transitioned to domestic funding first, whilst those that are a global CGH receive longer term external funding. Implementing this approach would have several advantages. First, it would create a mechanism to ensure governments of high-risk countries are held accountable for fulfilling their domestic funding commitments. By actively monitoring and evaluating their financial contributions to polio eradication efforts, it becomes easier to identify any discrepancies or shortcomings, enabling necessary interventions to be taken. This accountability not only helps in sustaining polio eradication efforts but also promotes transparency and good governance. Additionally, this approach would facilitate the mobilization of global resources for sustaining polio eradication and simultaneously strengthening health systems’ resilience. By providing a clear framework and demonstrating the interconnections between polio eradication and overall health system functioning, it becomes easier to garner support and resources from international organizations, donors and other stakeholders. This collective effort ensures a comprehensive approach to both sustaining eradication and strengthening the broader health systems necessary for effective disease prevention and control. Moreover, linking domestic funding commitments with global resources creates a more sustainable and coordinated approach. It encourages governments to prioritize investments in health systems, acknowledging the importance of building resilient systems that can effectively respond to public health emergencies beyond polio. This holistic approach could benefit both eradication efforts and the overall health outcomes of the population.

In conclusion, this analysis and the findings presented here highlight several potential areas for further research, both at the country and global levels. These findings should serve as a catalyst for new analyses that can deepen our understanding of the complexities of polio transition. In addition, they can provide valuable insights for policymakers to help shape the transition process itself.

## Supplementary Material

czad093_Supp

## Data Availability

The data underlying this article are available in the article and in its online supplementary material.

## Footnotes

1. Public goods are non-rival and non-excludable, i.e. multiple people can use them and nobody is excluded from benefit from them.

2. Information on the Pandemic Fund can be found here: https://bit.ly/3WmLQJs

## Reflexivity statement

This manuscript deals with a global issue pertaining mainly to LMICs and global donors’ financing polio eradication. The study was undertaken jointly by academics and practitioners at the WHO, thereby ensuring diverse inputs into the manuscript by appropriate stakeholders. Furthermore, the manuscript is co-authored by scholars at different levels of seniority: S.V. holds an MD degree and is pre-doctoral research fellow at the University of Milan and a MSc canditate in Epidemiology at the London School of Hygiene and Tropical Medicine; F.T. is a senior academic; D.L. holds an MSc and is a junior WHO official; C.P. and E.E. are senior WHO officials.

## References

[R1] Cassidy R, Borghi J, Semwanga AR et al. 2022. How to do (or not to do)…using causal loop diagrams for health system research in low and middle-income settings. *Health Policy and Planning* 37: 1328–36. 35921232 10.1093/heapol/czac064 PMC9661310

[R2] De Savigny D, Blanchet K, Adam T. 2017. *EBOOK: Applied Systems Thinking for Health Systems Research: A Methodological Handbook*. London, England: Open University Press, McGraw-Hill Education (UK).

[R3] Government of Syrian Arab Republic . 2021. Country Strategic Plans on Polio Transition: Integrated Public Health Teams – Syrian Arab Republic. Damascus, Syrian Arab Republic. 30 September 2021.

[R4] Government of the Republic of the Sudan . 2021. Country Strategic Plans on Polio Transition: Integrated Public Health Teams – Country Update – Sudan, Khartoum, Republic of the Sudan. 18 March 2021.

[R5] Groce NE, Banks LM, Stein MA. 2021. The Global Polio Eradication Initiative—polio eradication cannot be the only goal. *The Lancet Global Health* 9: e1211. doi: 10.1016/S2214-109X(21)00314-4 34416208

[R6] Marten R, Shroff ZC, Hanson K et al. 2022. Reimagining health systems as systems for health. *BMJ.* 379: o3025. 10.1136/bmj.o3025 36526278

[R7] Neel AH, Svea C, Catherine V et al. 2021. 30 years of polio campaigns in Ethiopia, India and Nigeria: the impacts of campaign design on vaccine hesitancy and health worker motivation. *BMJ Global Health* 6: e006002. 10.1136/bmjgh-2021-006002 PMC8336205 34344665

[R8] Saxenian H, Alkenbrack S, Freitas Attaran M et al. 2022. Sustainable financing for Immunization Agenda 2030. *Vaccine* Dec 1:S0264-410X(22): 01450–5 doi: 10.1016/j.vaccine.2022.11.037. 36464542

[R9] Shroff Z, Marten R, and Hanson K 2022a. Systems for health: everyone has a role. Flagship report of the Alliance for Health Policy and Systems Research. Geneva: World Health Organization.

[R10] Shroff ZC, Sparkes S, Skarphedinsdottir M, Hanson K. 2022b. Rethinking external assistance for health. *Health Policy and Planning* 37: 932–4. 35362537 10.1093/heapol/czac030 PMC9347018

[R11] Sim SY, Watts E, Constenla D, Brenzel L, Patenaude BN. 2020. Return on investment from immunization against 10 pathogens in 94 Low- and middle-income countries, 2011–30. *Health Affairs* 39: 1343–53. 32744930 10.1377/hlthaff.2020.00103

[R12] Tebbens RJD, Pallansch MA, Cochi SL et al. 2010. Economic analysis of the global polio eradication initiative. *Vaccine* 29: 334–43. 21029809 10.1016/j.vaccine.2010.10.026

[R13] Thompson K, Kalkowska D, Badizadegan K. 2022. Polio health economics: assessing the benefits and costs of polio, non-polio, and integrated activities of the Global Polio Eradication Initiative [version 1; peer review: 2 approved]. *Gates Open Research* 6: 5. doi: 10.12688/gatesopenres. PMC8881365 35280345

[R14] TIMB . 2017a. The end of the beginning. First Report of the Polio Transition Independent Monitoring Board. https://polioeradication.org/wp-content/uploads/2017/07/TIMB_Report-no1_Jul2017_EN.pdf, accessed 15 November 2022.

[R15] TIMB . 2017b. One door closes, another opens. Second Report of the Polio Transition Independent Monitoring Board. https://polioeradication.org/wp-content/uploads/2017/12/Second-TIMB-Report-December-2017-171218-en.pdf, accessed 15 November 2022.

[R16] TIMB . 2021a. Building stronger resilience. THE ESSENTIAL PATH TO A POLIO-FREE WORLD. Fifth Report of the Polio Transition Independent Monitoring Board. https://polioeradication.org/timb-members/, accessed 15 November 2022.

[R17] TIMB . 2021b. Navigating complexity. Fourth Report of the Polio Transition Independent Monitoring Board. https://polioeradication.org/wp-content/uploads/2021/02/4th-TIMB-Report-Navigating-Complexity-20210131.pdf, accessed 15 November 2022.

[R18] WHO . 1988. World health assembly global eradication of poliomyelitis by the year 2000. *WHA Resolution no WHA41*. Geneva, Switzerland: World Health Organization.

[R19] WHO . 2017. Polio transition planning. SEVENTIETH WORLD HEALTH ASSEMBLY, A70/14 Add.1. 19 May 2017. Geneva, Switzerland: World Health Organization.

[R20] WHO . 2018. Polio transition and post-certification. SEVENTY-FIRST WORLD HEALTH ASSEMBLY, A71/9. 24 April 2018. Geneva, Switzerland: World Health Organization.

[R21] WHO . 2021. *Financing common goods for health.* World Health Organization. https://www.who.int/health-topics/common-goods-for-health#tab=tab_1, accessed 28 December 2022.

[R22] WHO . 2022a. Global Polio Eradication Initiative Investment Case 2022-2026: investing in the promise of a polio-free world. Geneva, Switzerland: World Health Organization.

[R23] WHO . 2022b. Role of the polio network in COVID-19 vaccine delivery and essential immunization, Lessons learned for successful transition. Geneva, Switzerland: World Health Organization. Licence: CC BY-NC-SA 3.0 IGO.

[R24] WHO . 2022c. *WHO, Polio Transition Programme – Monitoring and evaluation dashboard.* World Health Organization. https://www.who.int/teams/polio-transition-programme/monitoring-and-evaluation-dashboard, accessed 26 December 2022.

[R25] WHO . 2023. Poliomyelitis. Polio transition planning and polio post-certification. *A76/14.* Geneva, Switzerland: World Health. Organization.

[R26] World Health Organization . 2022. Mid-term evaluation of the implementation of the Strategic Action Plan on Polio Transition (2018–2023). Corporate evaluation commissioned by the WHO Evaluation Office. EuroHealthGroup.

[R27] Yamey G, Jamison D, Hanssen O, Soucat A. 2019. Financing global common goods for health: when the world is a country. *Health Systems & Reform* 5: 334–49. 31860402 10.1080/23288604.2019.1663118

[R28] Yazbeck AS, Soucat A. 2019. When both markets and governments fail health. *Health Systems & Reform* 5: 268–79. 31684822 10.1080/23288604.2019.1660756

