# Supplementary material for: Leveraging global investments for polio eradication to strengthen health systems’ resilience through transition
Source: Health Policy Plan. 2024 Jan 23;39(Suppl 1):i93–i106. doi: 10.1093/heapol/czad093 (PMC10977911; doi:10.1093/heapol/czad093)
Supplement: czad093_Supp [file czad093_supp.zip › Supplemetary materials_160123 edited.docx]

**Supplementary materials**

**Table S1** List of 10 AFR polio high-risk countries that continue to receive support from the GPEI, with a view to full transition as of 2024

| **Countries** |
| --- |
| Angola, Chad, Democratic Republic of the Congo, Guinea, Ethiopia, Kenya, Nigeria, Niger, South Sudan |

| **Box S1 Polio transition during the COVID-19 pandemic**  In early 2020, at the beginning of the COVID-19 pandemic, the Polio Oversight Board (POB) of GPEI recommended “that all polio outbreak response, supplementary immunization activities (SIAs) be suspended until June 2020 and all preventive SIAs be postponed until the second half of the year”. This resulted in 28 countries suspending 62 polio vaccine SIAs. Routine polio immunization was also affected. Planned polio transition activities, including implementation of national polio transition plans, also slowed down or even stalled during the pandemic. It is estimated that 61% of the polio workforce spent at least 50% of their time on the COVID-19 response, in SEAR almost 2,600 personnel spent between ¼ and ¾ of their time on the COVID-19 response and in EMR 1,243 polio staff contributed to the pandemic response. Despite the circumstances, the estimated coverage of polio vaccinations (IPV1 and POL3) and measles containing vaccine (MCV1) remained more or less stable in the 7 polio transition priority countries in AFR in 2020, whereas AFP surveillance took a noticeable decline. Polio transition priority countries of SEAR appear to have been more affected by the pandemic in terms of coverage of polio and MCV, except Bangladesh, and a declining performance on AFP surveillance. COVID-19 provided an important opportunity to demonstrate how the polio infrastructure can support broader health security outcomes. Collaboration and coordination between the polio programme and other departments of WHO, particularly the health emergency division, has also been strengthened due to the pandemic response. The importance of the polio infrastructure to the COVID-19 response has been well documented by WHO[13]. |
| --- |

***Source:*** *EuroHealthGroup. 2022. Mid-term evaluation of the implementation of the Strategic Action Plan on Polio Transition (2018–2023). Corporate evaluation commissioned by the WHO Evaluation Office.*

| **Box S2. Comparison of GPEI, Gavi, and GFTAM phase-out strategies**   \|  \| **GPEI** \| **GFATM** \| **Gavi** \| \| --- \| --- \| --- \| --- \| \| **Eligible countries** \| 43^☨^ \| 133^*^ \| 77^#^ \| \| **Transition phases** \| None.  Every country can identify polio transition phases in coordination with local and international partners, even though it is not required. \| None.  GFATM can grant transition funding provided that some criteria are met. \| There are four phases:   \| **Ph1** \| Initial self-financing^†^ \| \| --- \| --- \| \| **Ph2** \| Preparatory transition^§^ \| \| **Ph3** \| Accelerated transition^¶^ \| \| **Ph4** \| Fully self-financing^‡^ \|   During the Ph4, for 5 years, countries can have access to Gavi vaccine tenders and can further apply for a 1-year financial support for HSS if not previously received. \| \| **Monitoring transition** \| WHO’s Monitoring and Evaluation framework for priority countries, with few process indicators. \| Regular updates every 6-12 months that include a thematic section based on strategic and implementation key performance indicators and a financial one with a minimum set of reliable information on the implementation of grants.^@^ \| The transition assessment is made for countries in phase 1, about 2-3 years before phase 2, and monitors the level of Penta3 coverage (90% threshold). \| \| IMB \| Yes, Transition Independent Monitoring Board (TIMB) \| Yes \| No \|  \| Long-term support \| Not foreseen, although it is recognized that long-term support to fragile countries will continue for specific activities (e.g., surveillance). \| Can assist countries to produce medicines and health products through GFATM’s procurement and Wambo platforms. ^£^ Some countries have existing arrangements to leverage the Pooled Procurement Mechanism to buy medicines with their own resources. \| Some countries have experienced programmatic shortcomings (e.g., insufficient institutional capacity). Gavi has committed to systematic engagement with countries post-transition (e.g., allocation of US$ 30 million for time-limited support). \| \| --- \| --- \| --- \| --- \|  \| ^☨^ Among those, 2 are endemic countries, 33 has active WPV and/or VDPV outbreaks, and 8 are considered to be at risk for polio emergence because of low immunization and surveillance levels based on GPEI website. Available at <https://polioeradication.org/> (Accessed 14 January 2023). ^*^For year 2020 based on data from the historical eligibility database of the GFATM. Available at: <https://www.theglobalfund.org/en/applying-for-funding/understand-and-prepare/eligibility/> (Accessed 26 December 2022). ^#^Up to 2018 data, based on the database for Gavi’s commitments, approvals & disbursements. Available at: <https://www.gavi.org/programmes-impact/our-impact/disbursements-and-commitments> (Accessed 26 December 2022). ^†^Low-income countries with a Gross National Income (GNI) per capita below US$ 1,025. ^§^Low-middle-income countries with GNI per capita between US$ 1,025 and 1,630. ^¶^Low-middle-income countries above the Gavi eligibility threshold (i.e., GNI per capita above US$ 1,630) can receive Gavi funds for up to 5 years. ^‡^Countries with a GNI per capita above US$ 1,630 that have stayed 5 years in Ph3 are now graduated from Gavi financing. ^@^Progress Update and Disbursement Request Form Instructions. Available at: <https://www.theglobalfund.org/media/11754/fundingmodel_pudr_instructions_en.pdf> (Accessed 26 December 2022). ^£^ Wambo.org is a digital procurement platform launched in 2016 to provide grant implementers with access to competitive prices, increased transparency, and reliability in the supply of quality health products. Since its launch in 2016, Wambo.org platform has been used to operationalize Pooled Procurement Mechanism orders and to bring together buyers and suppliers of health products. The product offering through Wambo has diversified each year. As of December 2020, the number of users had increased to 1,900, from over 170 organizations in 80 countries.  **Abbreviations**: GFATM=Global Fund to Fight AIDS, Tuberculosis and Malaria; GPEI=Global Polio Eradication Initiative; HSS=Health System Strengthening; IMB=Independent Monitoring Board; WHO=World Health Organization. \| \| --- \| |
| --- | --- | --- | --- | --- | --- | --- | --- | --- | --- | --- | --- | --- | --- | --- | --- | --- | --- | --- | --- | --- | --- | --- | --- | --- | --- | --- | --- | --- | --- | --- | --- | --- | --- |
