# Supplementary material for: Leveraging global investments for polio eradication to strengthen health systems’ resilience through transition
Source: Health Policy Plan. 2024 Jan 23;39(Suppl 1):i93–i106. doi: 10.1093/heapol/czad093 (PMC10977911; doi:10.1093/heapol/czad093)

## Slide 1
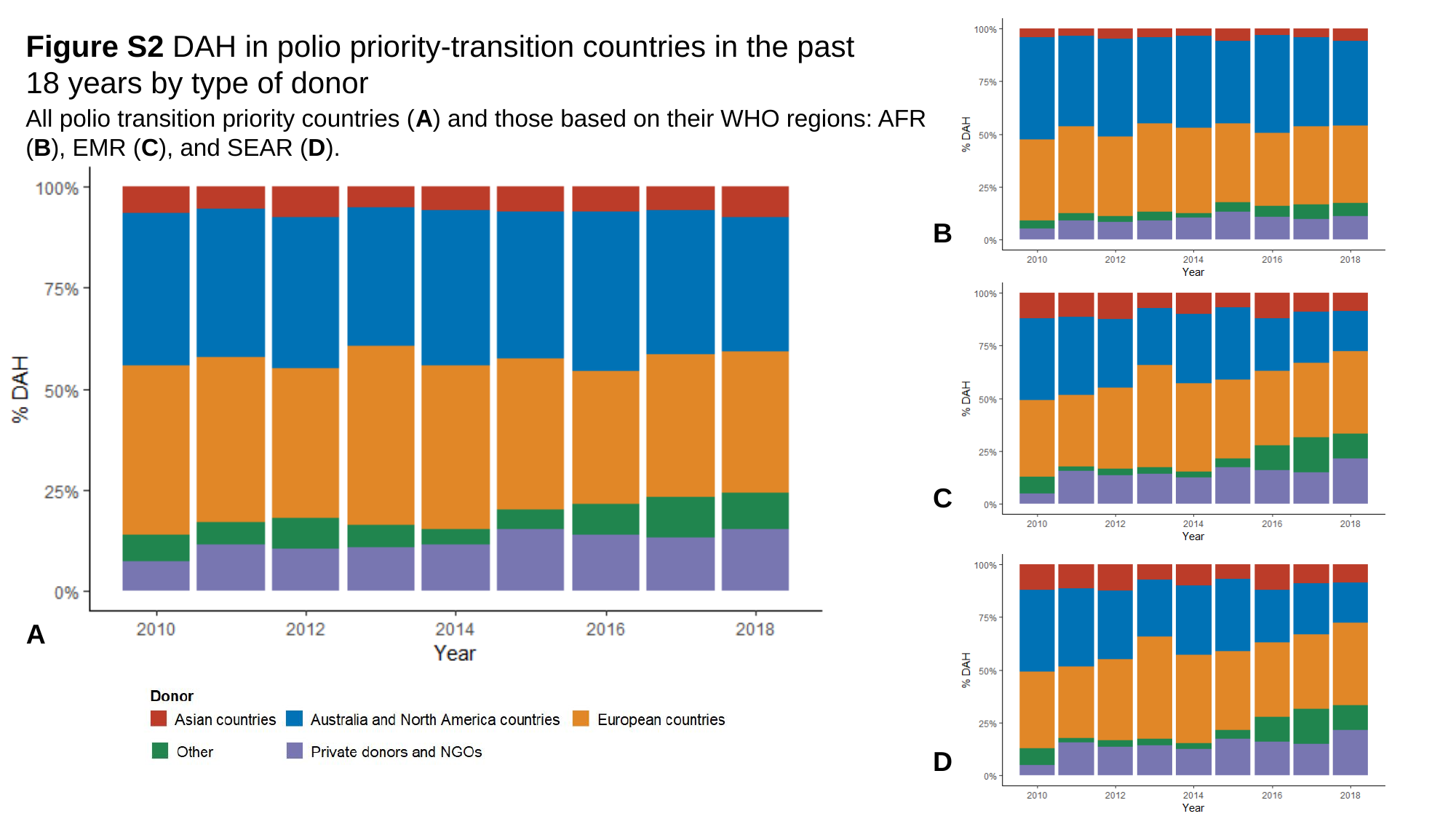

Figure S2 DAH in polio priority-transition countries in the past 18 years by type of donor
All polio transition priority countries (A) and those based on their WHO regions: AFR (B), EMR (C), and SEAR (D).
B
C
A
D

## Slide 2
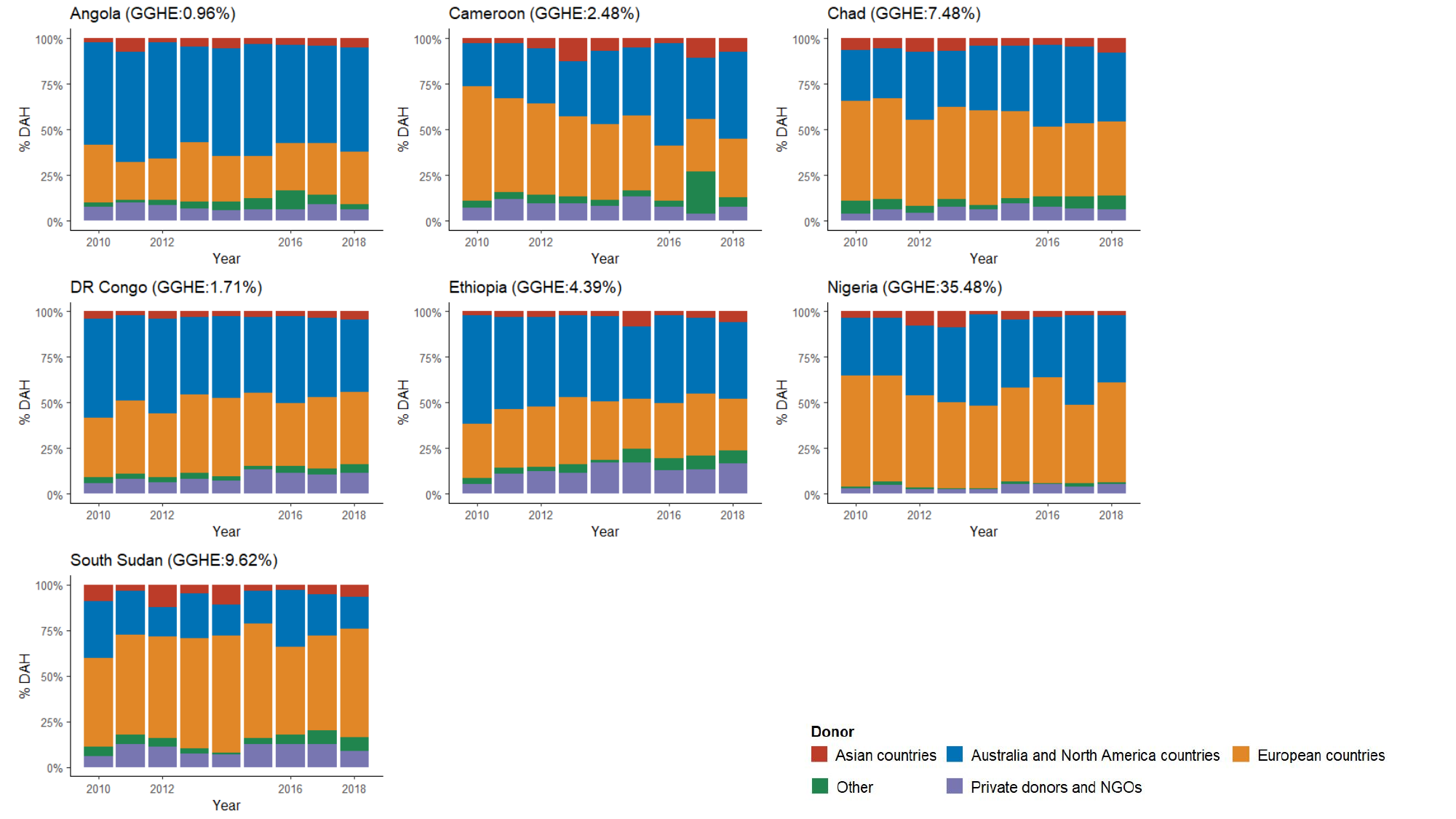

## Slide 3
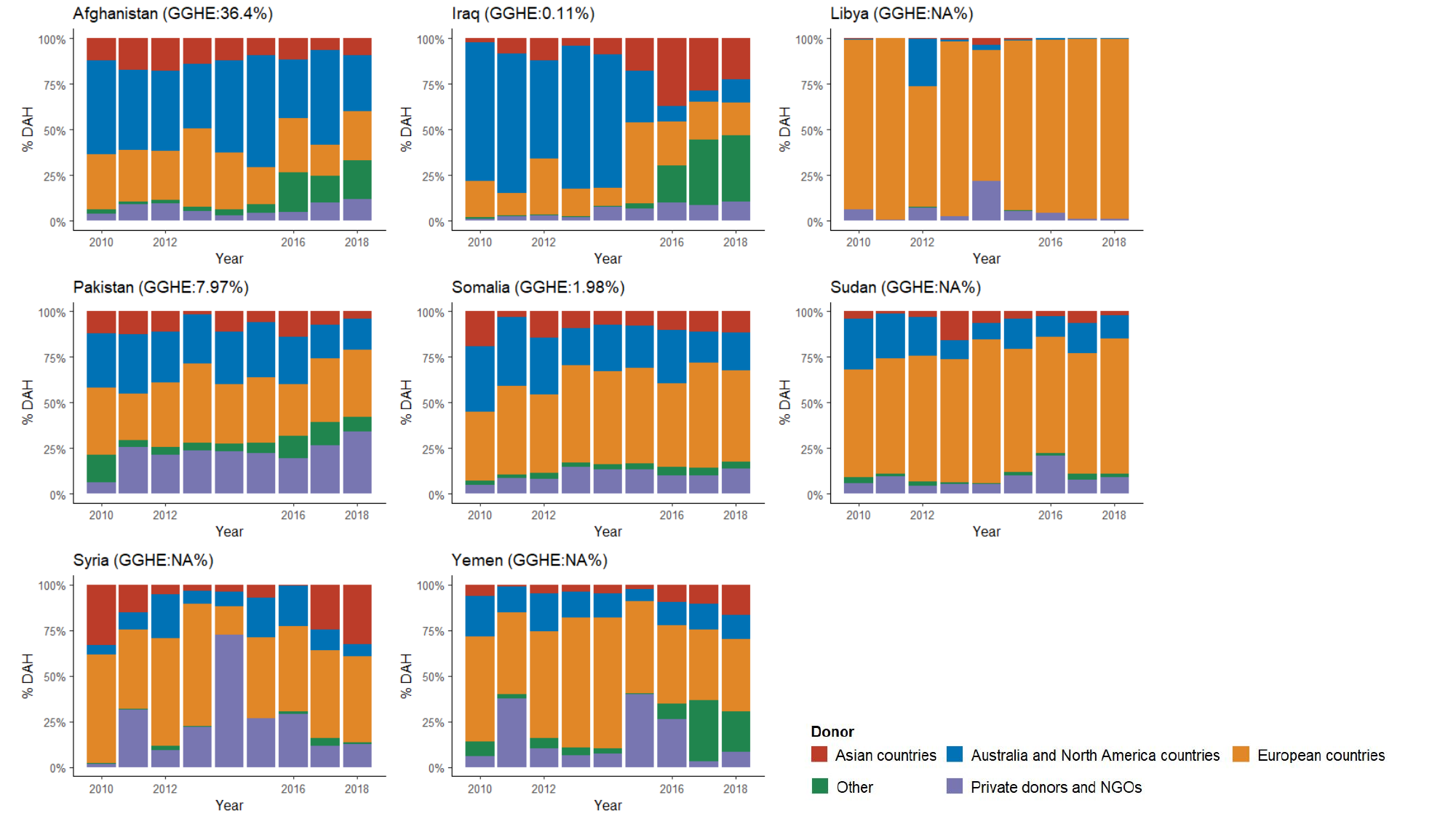

## Slide 4
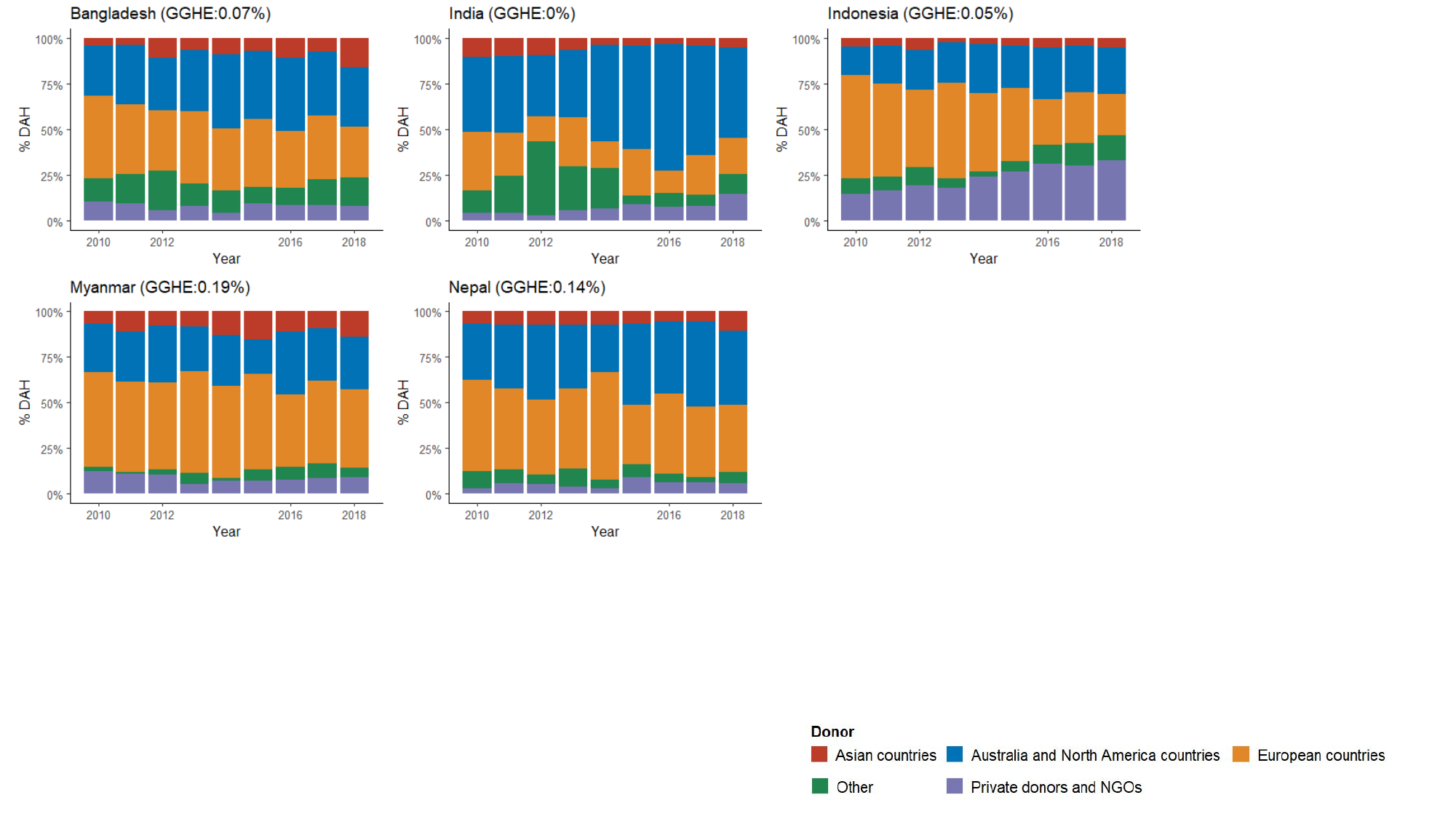

Supplement: czad093_Supp [file czad093_supp.zip › Figure S2 edited.pptx]
