# Supplementary material for: Leveraging global investments for polio eradication to strengthen health systems’ resilience through transition
Source: Health Policy Plan. 2024 Jan 23;39(Suppl 1):i93–i106. doi: 10.1093/heapol/czad093 (PMC10977911; doi:10.1093/heapol/czad093)
Supplement: czad093_Supp [file czad093_supp.zip › Figure S3.pptx]

## Slide 1
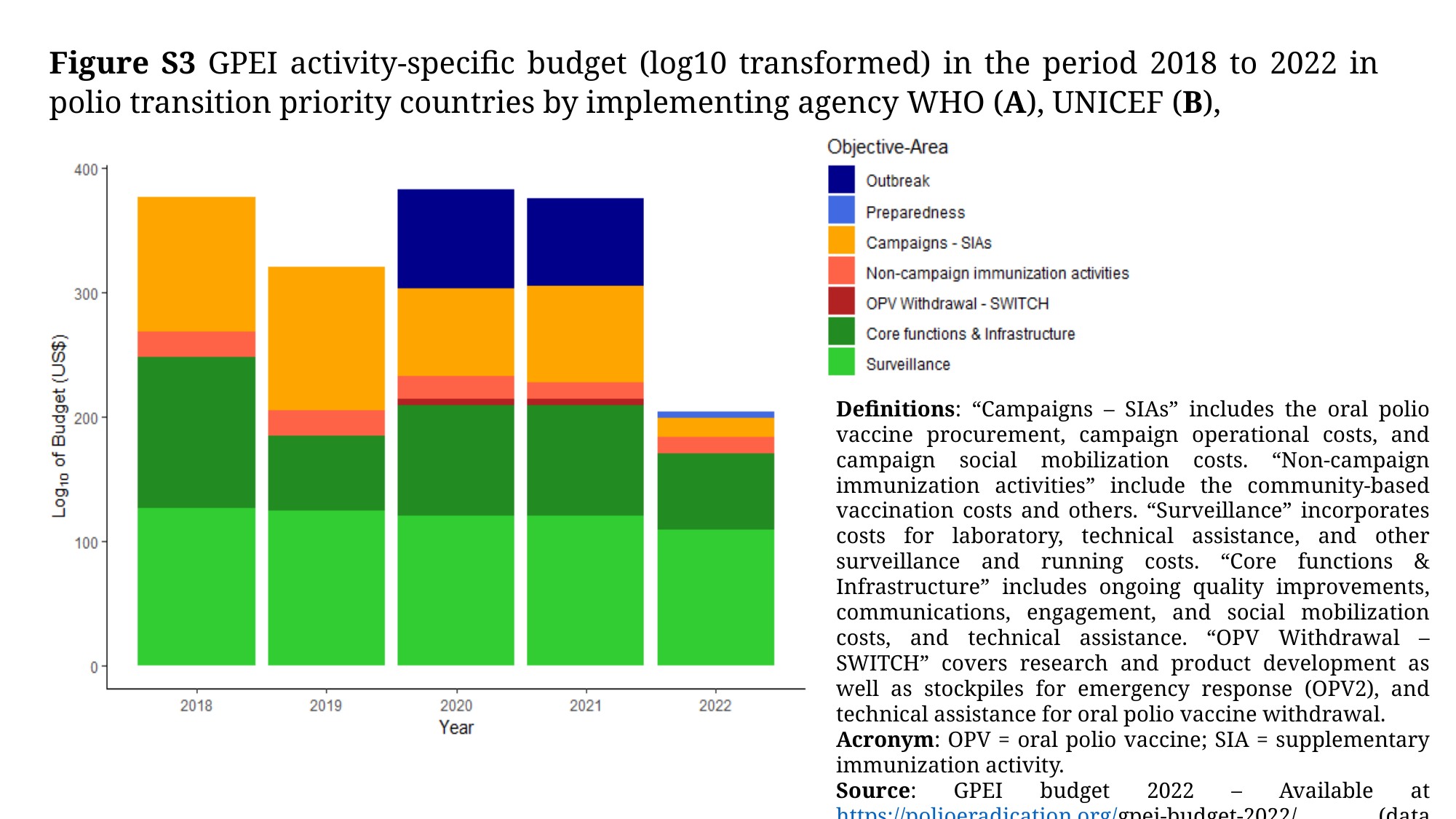

Figure S3 GPEI activity-specific budget (log10 transformed) in the period 2018 to 2022 in polio transition priority countries by implementing agency WHO (A), UNICEF (B),
Definitions: “Campaigns – SIAs” includes the oral polio vaccine procurement, campaign operational costs, and campaign social mobilization costs. “Non-campaign immunization activities” include the community-based vaccination costs and others. “Surveillance” incorporates costs for laboratory, technical assistance, and other surveillance and running costs. “Core functions & Infrastructure” includes ongoing quality improvements, communications, engagement, and social mobilization costs, and technical assistance. “OPV Withdrawal – SWITCH” covers research and product development as well as stockpiles for emergency response (OPV2), and technical assistance for oral polio vaccine withdrawal.
Acronym: OPV = oral polio vaccine; SIA = supplementary immunization activity.
Source: GPEI budget 2022 – Available at https://polioeradication.org/
gpei-budget-2022/ (data provided by GPEI).

## Slide 2
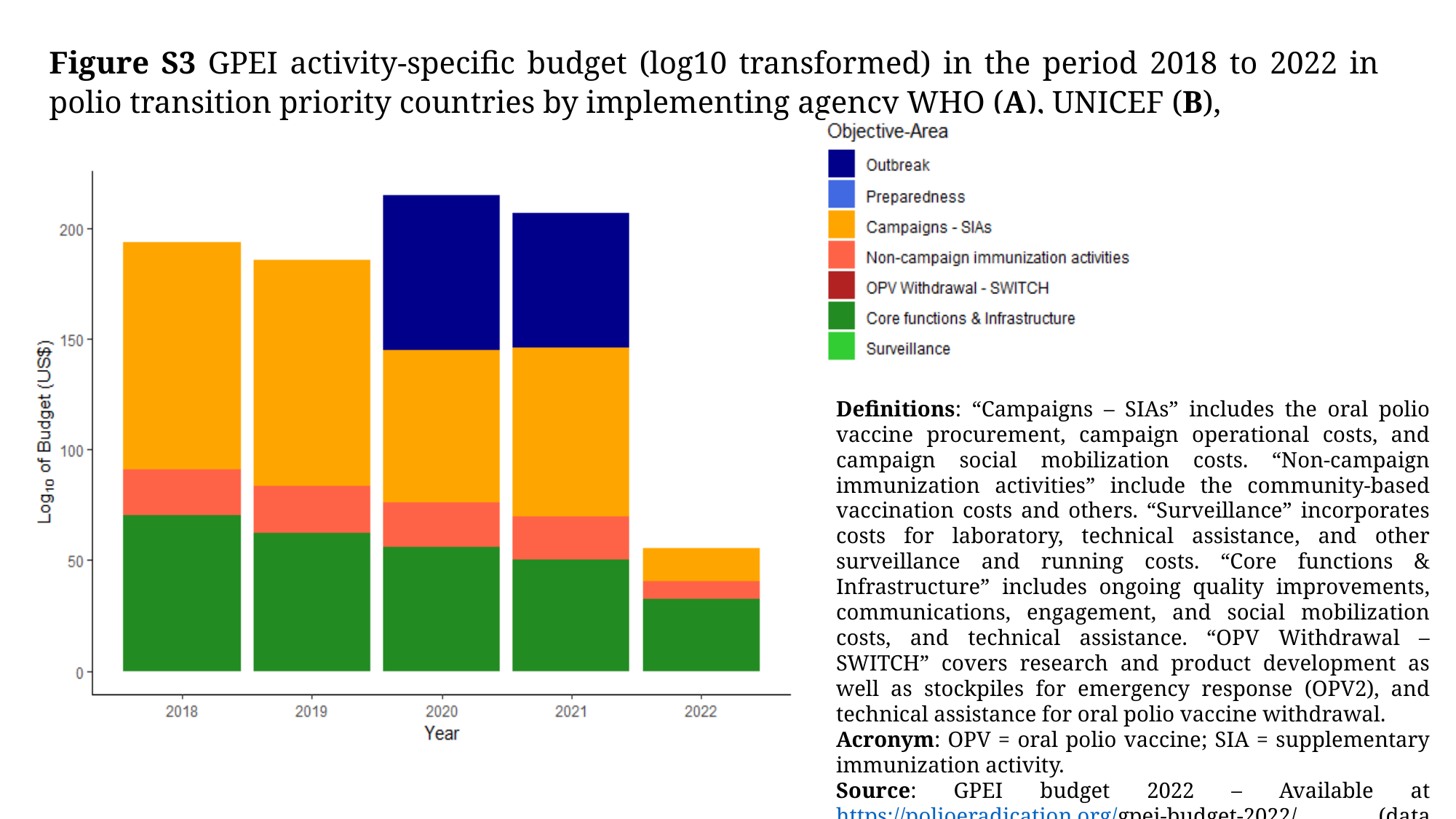

Figure S3 GPEI activity-specific budget (log10 transformed) in the period 2018 to 2022 in polio transition priority countries by implementing agency WHO (A), UNICEF (B),
Definitions: “Campaigns – SIAs” includes the oral polio vaccine procurement, campaign operational costs, and campaign social mobilization costs. “Non-campaign immunization activities” include the community-based vaccination costs and others. “Surveillance” incorporates costs for laboratory, technical assistance, and other surveillance and running costs. “Core functions & Infrastructure” includes ongoing quality improvements, communications, engagement, and social mobilization costs, and technical assistance. “OPV Withdrawal – SWITCH” covers research and product development as well as stockpiles for emergency response (OPV2), and technical assistance for oral polio vaccine withdrawal.
Acronym: OPV = oral polio vaccine; SIA = supplementary immunization activity.
Source: GPEI budget 2022 – Available at https://polioeradication.org/
gpei-budget-2022/ (data provided by GPEI).
