# Supplementary material for: Leveraging global investments for polio eradication to strengthen health systems’ resilience through transition
Source: Health Policy Plan. 2024 Jan 23;39(Suppl 1):i93–i106. doi: 10.1093/heapol/czad093 (PMC10977911; doi:10.1093/heapol/czad093)
Supplement: czad093_Supp [file czad093_supp.zip › Table S2_new.docx]

**Table S2** List of references grouped by polio transition priority country about their key polio functions

| **Country** | **References ^[[1]](#footnote-1)^** |
| --- | --- |
| Angola | - Plano de Transição da Pólio de Angola 2019-2024 (2019) - Mission Report for the Polio Transition Plan of Angola (2019, draft) - Joint external evaluation of IHR core capacities of Angola (2019) - Morais et al. Genetic and epidemiological description of an outbreak of circulating vaccine-derived polio-virus type 2 (cVDPV2) in Angola, 2019-2020. Vaccine 2023;41 Suppl 1:A48-A57 doi: 10.1016/j.vaccine.2023.02.035 (2023) |
| Cameroon | - Plan de Transition du Programme d'eradication de la Poliomyelite au Cameroon 2017-2021 (2017) - WHO Polio Transition Country Mission to Cameroon (2019) - Joint External Evaluation of IHR Core Capacities of the Republic of Cameroon (2017) |
| Chad | - Plan de Transition de la Poliomyelite du Chad 2018-2022 (2017) - Plan de Transition Polio 2022-2026 (2022) - WHO Polio Transition Country Mission to Chad (2019) - Joint External Evaluation of IHR Core Capacities of the Republic of Chad (2017) |
| DRC | - Plan de transition du Programme Mondial d’Eradication de la Polio RD Congo, 2022-2024 (2021) - Plan de transition du Programme Mondial d’Eradication de la Polio RD Congo, 2018-2022 (2017) - Évaluation externe conjointe des principales capacités RSI de la République démocratique du Congo (2018) |
| Ethiopia | - Federal Democratic Republic of Ethiopia Polio Program Transition Plan 2018-2022(2018) - Joint external evaluation of IHR core capacities of the Federal Democratic Republic of Ethiopia (2016) - WHO Polio Transition Country Mission to Ethiopia (2018) |
| Nigeria | - Nigeria Costed Polio Transition Plan 2020-2023 (2021, draft) - Joint external evaluation of IHR core capacities of the Federal Republic of Nigeria (2017) - Nigeria Polio Transition Business Case 2019 - 2023 (2019) - WHO Polio Transition Country Mission to Nigeria (2021) |
| South Sudan | - Transition Plan for the Polio Eradication Initiative of the Republic of South Sudan (2018) - Joint external evaluation of IHR core capacities of the Republic of South Sudan (2017) - WHO Polio Transition Country Mission to South Sudan (2019) |
| Iraq | - Polio Transition Update TIMB Interview with Chair, Sir Liam Donaldson (2021) - Joint external evaluation of IHR core capacities of the Republic of Iraq (2019) - Country Strategic Plan for Polio Transition – Iraq (2022) |
| Libya | - Joint external evaluation of IHR core capacities of Libya (2018) - Strategic Plan on Polio Transition – Libya (2022) |
| Somalia | - Transition Plan 2018-2019 for the Polio Eradication Initiative in Somalia (2018) - Draft Polio Transition Plan for Somalia, 2021-2024 (2021) - Joint external evaluation of IHR core capacities of the Republic of Somalia (2016) |
| Sudan | - Country Strategic Plans on Polio Transition: Integrated Public Health Teams – Sudan (2021) - Draft Sudan Polio Transition Plan 2018- 2030 (2018) - Joint external evaluation of IHR core capacities of the Republic of the Sudan (2016) |
| Syria | - Syrian Arab Republic Strategic Plan on Polio Transition: Integrated Public Health Teams (2021) |
| Yemen | - Yemen Polio Transition Plan (2021) |
| Bangladesh | - Polio Transition Plan Bangladesh (2018) - Joint external evaluation of IHR core capacities of the People's Republic of Bangladesh (2016) - Bangladesh government presentation at Montreux meeting - 13 November 2018 - WHO Polio Transition Country Mission to Bangladesh (2018) |
| India | - NPSP Transition Planning Framework 2018-2021 & 2022-2026 - WHO Polio Transition Country Mission to India (2018) |
| Indonesia | - Sustainability Planning for Maintaining Indonesia’s Polio-Free Status (2017) - Joint external evaluation of IHR core capacities of the Republic of Indonesia (2017) |
| Myanmar | - Draft Polio Transition Planning Myanmar (2017, draft) - Joint external evaluation of IHR core capacities of the Republic of the Union Myanmar (2017) |
| Nepal | - Nepal WHO Polio Asset Map (2015) - Polio Transition Planning Nepal (2017, draft) |

1. The various national transition plans are at different stages of development, government endorsement and revision. [↑](#footnote-ref-1)
